# Patient-Reported Social Impact of Molecularly Confirmed Macular Dystrophies and Cone–Rod Dystrophies

**DOI:** 10.3390/jcm14227928

**Published:** 2025-11-08

**Authors:** Nina Zehe-Lindau, Birgit Lindau, Heidi Stöhr, Bernhard H. F. Weber, Georg Spital, Ulrich Kellner

**Affiliations:** 1 Rare Retinal Disease Center, AugenZentrum Siegburg, MVZ Augenärztliches Diagnostik- und Therapiezentrum Siegburg GmbH, Europaplatz 3, 53721 Siegburg, Germany; ninaz0412@gmail.com (N.Z.-L.); b.lindau@osg.de (B.L.); 2 RetinaScience, Postcode 301212, 53192 Bonn, Germany; 3 Institute of Human Genetics, University of Regensburg, Franz-Josef-Strauss-Allee 11, 93053 Regensburg, Germany; heidi.stoehr@klinik.uni-regensburg.de (H.S.); bweb@klinik.uni-regensburg.de (B.H.F.W.); 4 Institute of Clinical Human Genetics, University Hospital Regensburg, Franz-Josef-Strauss-Allee 11, 93053 Regensburg, Germany; 5 Augenzentrum am St. Franziskus-Hospital Münster, Hohenzollernring 74, 48145 Münster, Germany; georg.spital@augen-franziskus.de

**Keywords:** macular dystrophy, cone–rod dystrophy, quality of life, patient-reported impact, social care, anxiety

## Abstract

**Objectives:** To identify patient-reported key disease-related challenges of macular and cone–rod dystrophies (MDs/CRDs) in a large consecutive cohort of individuals with molecularly confirmed diagnoses. **Methods:** Out of the 281 patients contacted, 194 (69.0%; 55.2% female) responded to an anonymized survey exploring the effects of MD/CRD on vocational training, professional careers, social participation, family life, personal well-being, and experience with ophthalmologic care. **Results:** While vocational training was generally less affected, professional careers were frequently disrupted, with 20.6% of patients aged ≥ 50 retiring early. A majority (54.7%) reported feeling restricted in public life. Financial constraints were noted by 20%. A negative impact on familial life (12.3%) was less frequently reported compared to anxiety (74.2%) and depression (15.8%). Diagnostic delays (≥2 years) were common (34.2%), along with a notable rate of initial misdiagnoses (22.1%). The lack of adequate psychological support was a major complaint in professional care. **Conclusions:** Compared to a previous study in retinitis pigmentosa, MD/CRD patients reported differing patterns of burden, especially in early retirement and family impact. Our findings underscore the need for ophthalmic and social care providers to accelerate the diagnostic process and enhance access to financial assistance and psychological support as key areas to improve patient care.

## 1. Introduction

Inherited macular dystrophies (MD) and cone–rod dystrophies (CRD) predominantly involve the posterior pole of the retina, affecting central visual functions like visual acuity, central visual field, and color vision [1,2]. Usually, disorders located within the temporal vascular arcades are considered as MDs, and those additionally affecting peripheral regions are called CRDs. However, there are differences between regions affected on ophthalmoscopy, retinal imaging, and functional testing; there is a continuous spectrum without distinct means of separation between MDs and CRDs, and during disease progression, several MDs will develop into CRDs [1,2,3]. MDs/CRDs account for approximately 30% of inherited retinal dystrophies (IRDs) in Germany [4]. In industrialized countries, IRDs are the leading cause of blindness in infants and adults under 65 years of age [5,6,7,8,9].

Similar to other IRDs, the onset of MDs/CRDs is highly variable even among individuals with a similar genetic background within a single family and can range from early infancy to over 50 years of age. Most patients, however, experience symptoms within the first decades of life. Progressive loss of visual acuity, central visual field defects, and glare may affect vocational training, professional careers, mobility, social life, and communication. Visual disability is associated with financial challenges, especially in IRDs, because of reduced income [10,11] and increased personal health costs [12]. Mental health can be affected in visually disabled patients and their family members with an increased frequency of anxiety and depression [13,14]. In addition, patients complain about the often lengthy process towards an established clinical and genetic diagnosis, as well as other insufficiencies in ophthalmic and psychological care [15].

In contrast to retinitis pigmentosa (RP), the patient-reported impact of MD/CRD has not been studied, with the exception of two small studies on Sorsby fundus dystrophy and Stargardt disease [16,17]. Usually, MD/CRD patients have been included within the group of all IRDs, often without consistent genetic confirmation [13,15,18].

The purpose of the present study was to address this gap by conducting a large anonymous survey focusing exclusively on patients with molecularly confirmed MD/CRD. The design of the study was similar to our previous study on patients with molecularly confirmed RP [19] to facilitate a direct comparison of the impact of different visual disabilities on the quality of life between MD/CRD and RP.

## 2. Materials and Methods

By searching two separate databases in two separate specialized centers, a consecutive series of 318 patients with molecularly confirmed cases of non-syndromic or syndromic MD or CRD was identified. Patients were eligible for inclusion if they had at least one pathogenic or likely pathogenic variant in autosomal dominant or X-linked MD/CRD or two pathogenic or likely pathogenic variants in a known gene for autosomal recessive MD/CRD; had been examined at one of the centers at least once between January 2018 and January 2024; were at least 18 years old at the time of the survey; and resided in Germany (the infrastructure of social help differs between European countries).

More than 60% of these patients had an initial diagnosis of MD, and most patients were of Caucasian ethnicity. Clinical assessments and molecular genetic confirmation were performed as previously described [20,21]. Ethics approval for the study was received from the respective ethics committees of the North Rhine (ID: 2024037, 27 February 2024) and Westphalia–Lippe (ID 2024-234-b-S, 9 April 2024) medical associations.

The concise, anonymous questionnaire was designed based on previously indicated patient concerns [13,15] and consisted of nine structured questions with predefined answer options (see Supplementary Materials). Aside from the disease terms (MD/CRD vs. RP), it was identical to the questionnaire used for a previous study on patients with molecularly confirmed RP [19], ensuring that anonymity, age, and age at onset were recorded in ten-year intervals. Seven questions addressed gender, vocational training, work experience, social life, family life, personal challenges, and ophthalmologic care.

With the aim of reaching patients in a broad age range and taking into account reduced visual function, the survey was conducted using a written questionnaire. To ensure anonymity, patients were contacted via postal mail, including a cover letter (explaining the study’s background, purpose, and the measures taken to ensure anonymity), an unmarked questionnaire, and a prepaid, pre-addressed, unmarked return envelope. Patients were required to respond to questions regarding age range, gender, and age at onset. For the remaining questions, either multiple responses or the option to skip the question was allowed. All survey letters were mailed on the same day, and patients had four weeks to respond. Patients were asked not to include any personal information, as this would be considered a breach of anonymity and would exclude their responses from the study.

Return letters were handled by two of the authors without access to the clinical databases. Questionnaires and envelopes were separated to delete traceable postal documentation prior to analysis of the questionnaires.

## 3. Results

### 3.1. Patient Characteristics

A questionnaire was sent to a consecutive series of 318 patients. A total of 37 out of 318 survey letters were returned due to invalid addresses, resulting in a final patient cohort of 281 individuals. Within the four-week response period, 194 out of 281 patients (69.0%) submitted completed questionnaires. No responses were excluded because of the inclusion of personal information. The distribution of age groups and gender among respondents is shown in Table 1. The majority (102 out of 194; 52.6%) were older than 50 years of age. In terms of gender, 107 respondents (55.2%) identified as female, 85 (43.8%) as male, and 2 (1.0%) as non-binary. Notably, male respondents tended to be younger, with 42.4% under 41 years of age, compared to 26.2% of female respondents in the same age group.

The distribution of reported disease onset is presented in Table 2. Most patients (102 out of 194, 52.6%) experienced initial symptoms within the first three decades of life. However, 73 patients (37.6%) noted their first symptoms after the age of 40. Gender differences were observed in the reported onset, with female patients most frequently experiencing onset in the fifth decade of life, whereas male patients most frequently indicated onset in the first decade.

### 3.2. Vocational Training

A total of 173 out of 194 patients (89.2%) provided responses regarding their vocational training experience (Table 3). Among the 21 non-respondents, 14 were over 50 years of age. The majority of respondents (141 out of 173; 81.5%) reported no issues during their planned vocational training, without a gender difference. Some patients (9 out of 173; 5.2%) had not yet started vocational training, including two individuals who were unable to begin their desired program. Additionally, 17 patients (9.8%) were unable to start their intended vocational training. Three patients had to terminate their first training program (1.7%). Eight patients reported a prolonged period of more than one year necessary for their vocational training compared to the initial planning. One patient had to change her vocational training more than twice.

### 3.3. Professional Life

A total of 157 out of 194 patients (80.9%) provided responses regarding their professional life experiences (Table 3). Six patients who had not yet started vocational training did not respond to this question. The majority (109 out of 157; 69.4%) reported no severe difficulties in their professional careers (67.4% of females, 72.5% of males). However, 28 patients (17.8%) had to retire early, including 5 individuals who were unable to start their professional careers despite completing vocational training. Among patients 40 years and older, 23 out of 130 (17.7%) had to retire early because of their MD/CRD. In patients 50 years and older, this proportion slightly increased, with 21 out of 102 (20.6%). Because of visual-related problems, nine patients had to change their careers more than twice. Fifteen patients (9.6%) experienced long-term unemployment lasting more than one year.

### 3.4. Social Disadvantages

A total of 190 out of 194 patients (97.9%) provided responses regarding social challenges (Table 3). A minority of 53 patients (27.9%) reported experiencing no social challenges. However, the majority noted limitations in public life participation as a consequence of their MD/CRD (104 patients; 54.7%). One fifth complained about financial restrictions (35 patients; 18.4%). Among those reporting financial difficulties, 15 out of 28 retired early, 9 out of 15 experienced unemployment for more than a year, and 3 out of 9 had to change careers more than twice, highlighting the impact of employment on financial independence. Additionally, 54 out of 194 patients (28.4%) received financial or other forms of support. Of these, 24 patients (44.4%) considered the support sufficient, while 30 patients (55.6%) found it insufficient, independent of age, disease duration, and gender. Notably, 8 out of 34 patients (23.5%) who reported financial restrictions did not receive any form of financial support.

### 3.5. Family Life

A total of 162 out of 194 patients (83.5%) provided responses regarding possible challenges in their family life (Table 3). A vast majority, 142 out of 162 patients (87.7%), reported no negative impact of MD/CRD on their familial situation, with a higher percentage among males (92.8%) compared to females (83.5%). This difference was independent of age. In seven cases, MD/CRD was cited as the reason for the end of a partnership. Additionally, 16 out of 162 patients (9.9%) reported that MD/CRD influenced their decision to forgo having children. This group included two individuals who otherwise reported no challenges in their familial life.

### 3.6. Personal Impairments

A total of 190 out of 194 patients (97.9%) provided responses regarding personal impairments (Table 3). Among them, only 23 patients (12.1%) reported feeling no personal impairments because of MD/CRD, without gender differences. The most frequently self-reported concern was anxiety about the future because of disease progression, affecting 141 out of 190 patients (74.2%), and it was slightly more frequent in females. Depression was self-reported by 30 patients (15.8%) and was slightly more frequent in males. Additionally, 80 patients (42.1%) described experiencing other personal impairments related to MD/CRD.

### 3.7. Ophthalmic Care

A total of 190 out of 194 patients (97.9%) provided responses regarding their ophthalmological care (Table 3). A slight majority, 114 out of 190 patients (60.0%), felt that their ophthalmologic and molecular genetic diagnosis was provided within an appropriate time frame. However, even among these, five patients (4.4%) still perceived a delay in their MD/CRD diagnosis. A marked delay between the initial ophthalmological examination due to MD/CRD symptoms and the confirmed diagnosis was reported by 65 out of 190 patients (34.2%). Major concerns regarding the diagnostic process included a delay of more than two years in 29 patients (15.3%) and more than five years in 36 patients (19.0%). Additionally, 42 patients (22.1%) experienced one or more misdiagnoses before receiving a confirmed MD/CRD diagnosis. More than one-third of the patients, 68 out of 190 (35.8%), expressed that they lacked psychological support throughout the progression of their disease.

### 3.8. Comparison with RP Patients

In a previous study using the same methodology, patients with molecularly confirmed retinitis pigmentosa (RP) were contacted and had a similar response rate (Table 4) [19]. Patient-reported impact was similar for RP and MD/CRD in most aspects; however, some differences need to be noted. In the RP study, patients were younger compared to the MD/CRD study. Disease onset was later in life in MD/CRD compared to RP. The difference in a later manifestation of MD/CRD compared to RP affected women more than men. Regarding vocational training, no gender difference was observed in MD/CRD, in contrast to a female advantage in RP. The response rate regarding professional life was lower in MD/CRD. Early retirement rates were higher in RP patients, especially in patients older than 50 years of age. Social support was more frequently perceived as sufficient by RP patients. The negative impact on family life was lower in MD/CRD, while the gender difference was similar. In contrast to RP, there was no gender difference regarding personal impairments in MD/CRD.

## 4. Discussion

To the best of our knowledge, this is the first study evaluating the patient-reported impact of MD/CRD on daily life in a large cohort of genetically confirmed MD/CRD patients. The response rate of 69.0% was similar to our previous study on RP patients and higher than that of a similar study on IRD patients [13,19]. This likely reflects the recruitment from two specialized IRD care centers, the concise format of the questionnaire, and the strong emphasis on anonymization.

Symptom onset of MD/CRD was observed by most patients in this study within the first three decades of life (Table 2). In comparison to RP, the onset of MD/CRD was generally later, and more patients noted onset after 40 years of age [19]. Similarly to RP, the impact on daily life was limited in the early stages, as they interfered with vocational training in only 13.8% of patients. Although personal career aspirations do not differ between individuals with and without visual impairment [22], it can be expected that visual dysfunctions influence patients to select vocational paths according to their personal abilities. A delay in vocational training contributes to reduced lifetime income [10].

The progression of MD/CRD is associated with increasing challenges in professional life. Some patients are unable to enter the workforce despite completing vocational training, while others are eventually forced to withdraw from employment. With increasing age, the likelihood of early retirement increased slowly (Table 3). In contrast to RP patients, the rates of early retirement were lower both in patients 40 years and older (MD/CRD: 17.7%, RP: 39.8%) and in patients 50 years and older (MD/CRD: 20.6%, RP: 50.0%) [19]. Early loss of central vision in MD/CRD would be expected to interfere with many professional tasks. The lower likelihood of early retirement in MD/CRD patients may reflect the beneficial effects of modern low-vision aids and rehabilitation programs that support continued employment despite central vision loss, greater occupational flexibility, or a later onset of disease in MD/CRD patients. In contrast, extended periods of unemployment because of delayed vocational training or difficulties securing employment were similar between MD/CRD (9.6%) and RP (13.3%). Unemployment because of visual impairment further contributes to reduced lifetime income [10,23].

Social challenges as a consequence of MD/CRD manifest primarily as reduced participation in public life (54.7%). Social isolation has been observed in more than half of individuals in previous IRD studies [13,24]. The risk of bumping into people or objects, challenges with stairs, and an increased likelihood of falls limit mobility and lead to self-imposed isolation [25,26]. Reduced activity and other behavioral changes have been observed already in children with visual impairment [27,28]. Syndromic MD/CRD most likely has an additional impact on participation in public life [29], but it has not been specifically studied in MD/CRD-associated syndromes.

Financial restrictions because of MD/CRD (18.4%) had a similar frequency as in our RP study (19.4%), but they were less frequent compared to other IRD studies (44%) [13]. Approximately one-third of patients received financial aid or other forms of support. In contrast to RP patients [19], the majority expressed dissatisfaction with the level of support received, and 23.5% of those reporting financial difficulties did not receive any assistance. This underscores the need to thoroughly evaluate and improve social and financial support systems to better address accessibility and patients’ needs in all disorders associated with impaired visual function [30,31].

Similar to RP, nearly 80% of patients reported no challenges in their family life, although it can be expected that financial challenges affected personal and familial circumstances. One tenth of the patients chose not to have children because of their MD/CRD. Many of these decisions were likely made prior to molecular genetic testing, underscoring the importance of timely genetic testing and appropriate counseling.

Anxiety and depression are frequently reported in association with IRDs [13,24,32,33,34]. In the present study, 74.2% of patients self-reported anxiety about their future situation. Previous studies on RP and IRD have reported anxiety rates ranging from 65.6% to 86% [13,19,25,35]. Similar to detailed anxiety assessments in RP [32,36,37], anxiety rates were independent of patient age or disease duration.

Depression suspected as a consequence of MD/CRD was self-reported by 15.8% of patients (RP patients, 24.2%) [19], which was lower compared to studies on non-syndromic (65%) or syndromic RP (86.2%) [13,38]. It is important to note that these numbers are based on patient-reported diagnoses, without formal psychological verification. Different methods have been used to estimate the frequency and severity of depression in RP and other ocular disorders (e.g., ICD-10 codes, validated questionnaires, HADS, BDI) [26,39,40,41]. This variation in methodology probably affects the frequency of the diagnosis. Restricted access to psychological services and individual reluctance to seek such care most likely contribute to an underestimation of clinically confirmed anxiety and depression. Consequently, the observed self-reported rates underscore an important unmet need warranting further investigation.

Determining whether visual impairment itself or the resulting unemployment primarily drives depressive symptoms remains challenging. Pre-existing depression may increase the risk of unemployment in patients with visual impairment. While RP patients with depression have lower employment rates [39], unemployment is associated with higher rates of depression [33]. Depression associated with visual impairment is common across a wide range of ophthalmic disorders [42,43,44]. Depression itself can exacerbate financial difficulties and negatively impact social life [45].

For decades, IRD patients have complained about the difficulties of the patient journey, including the time delay between symptom onset and clinical diagnosis, the frequency of initial misdiagnosis, and the lack of psychological support after establishing the diagnosis and throughout disease progression. In our studies, 34.2% of MD/CRD patients and 28.6% of RP patients reported a diagnostic delay of more than two years. Though one would hope that the widespread availability of retinal imaging and novel methods facilitating extensive genetic testing would shorten the diagnostic process, recent studies still emphasize the need to further improve the patient pathway in diagnosing IRDs [15,46,47].

The frequency of misdiagnosis is difficult to establish. It is nearly impossible for patients to distinguish between true diagnostic errors (e.g., mistaking MD/CRD for acquired disorders) and variations in terminology used by different ophthalmologists (e.g., macular dystrophy, Stargardt disease, cone–rod dystrophy, and ABCA4 gene-associated MD/CRD). Unfortunately, the lengthy process to reach a definitive diagnosis suggests that many patients did at least receive one incorrect diagnosis. The diagnostic guidelines for IRDs were recently revised in Germany [48]. However, raising awareness among healthcare professionals about the importance of early and accurate diagnosis for IRD patients will remain a continuous task for all healthcare providers associated with IRDs. In addition, discipline-specific directives and guidelines for the clinical and genetic diagnostic process would reduce the psychological burden associated with unexplained visual loss.

More than one-third of MD/CRD and RP patients did not receive psychological support after diagnosis and throughout the progression of the disease. Continuous psychological support should be an integral part of patient care, provided by a multidisciplinary team that considers the patient experience [34,49,50,51,52,53,54]. Such support could reduce the frequency of anxiety and depression and improve patients’ social interactions and work capabilities. At least in Germany, the number of psychological support providers experienced with IRD patients is low. An interdisciplinary exchange between separate healthcare providers involved in the care of IRD patients will be important to establish uninterrupted patient care. As a possible advantage for the social care system, improved diagnostic processes, rehabilitation, and patient care should have an impact on the high healthcare and societal costs of IRDs [55,56].

The present study has several limitations. Firstly, the limitation of including patients with genetically confirmed MD/CRD excludes approximately 30% of MD/CRD patients for whom a genetic cause cannot yet be defined. Molecular genetic testing is usually initiated at specialized centers for rare retinal disease in Germany [53]; therefore, the study population may represent individuals who either had the good fortune or personal motivation to pursue their diagnosis to access these centers.

Secondly, this study employed a concise, anonymized, and non-validated questionnaire to assess the impact of MD/CRD on various aspects of patients’ lives. A more detailed analysis, especially of anxiety and depression, with a validated methodology, would be desirable. However, these methodologies are more time-consuming and often require direct personal communication [36]. This limits the number of study patients due to research resources, as well as the availability of patients for longer test periods or for traveling to research centers. This might induce a different bias in patient selection in more detailed studies.

Thirdly, to preserve anonymity, the survey did not collect detailed personal comments. While this approach enabled the inclusion of a large patient cohort, it limited the depth of individual responses. In addition, a comparison with clinical or molecular genetic findings, as well as a detailed statistical analysis of contributing factors, was not possible. Fourthly, because of the anonymity of the survey, we cannot exclude the possibility that some patients were contacted and responded twice because they were examined at both participating centers. However, given the geographical distance between the two centers, the previous experience of having patients present to both centers and all return letters pre-addressed to one single address, few duplicate responses are expected and should not impact the study’s findings.

Finally, there possibly is a difference between the experience of patients born into a family with other affected family members versus patients without other affected family members. For the latter and the family, all experiences associated with MD/CRD are novel. Because of anonymization, we could not evaluate this aspect in the present study, but this aspect should be included in future research.

## 5. Conclusions

The present study provides the first overview of the current challenges faced by patients with genetically confirmed MD/CRD in Germany. Future research should focus on a more detailed analysis of the quality of life in correlation with visual function and clinical phenotype, the professional confirmation of anxiety and depression with validated psychometric tools, and the definition of standards for psychological care in MD/CRD patients.

The patient-reported impact of disease is comparable between MD/CRD and RP patients. Both our studies emphasize a continuous need to enhance a time-efficient diagnostic process for IRD patients. A confirmed diagnosis is mandatory for initiating rehabilitation, psychological support, and financial assistance. Beyond clinical and genetic diagnosis, a multidisciplinary approach for a lifelong, individualized patient pathway needs to be established and continuously re-evaluated. Societies and social care systems may benefit from improved care strategies by reducing increased primary healthcare and high secondary societal costs of IRDs.

## Figures and Tables

**Table 1:** Distribution of patient age.

Age Distribution	All		Female		Male	
Years	*n* = 194 *	%	*n* = 107	%	*n* = 85	%
18–20	10	5.2	5	4.7	5	5.9
21–30	31	16.0	12	11.2	19	22.4
31–40	23	11.9	11	10.3	12	14.1
41–50	28	14.4	17	15.9	10	11.8
51–60	48	24.7	28	26.2	20	23.5
61–70	36	18.6	25	23.4	11	12.9
>70	18	9.3	9	8.4	8	9.4

* Two respondents identified themselves as non-binary.

**Table 2:** Distribution of patient-reported age of onset.

Onset Distribution	All		Female		Male	
[Years of Age]	*n* = 194 *	%	*n* = 107	%	*n* = 85	%
0–10	44	22.7	15	14.0	29	34.1
11–20	32	16.5	19	17.8	12	14.1
21–30	26	13.4	15	14.0	11	12.9
31–40	19	9.8	10	9.4	9	10.6
41–50	41	21.1	30	28.0	11	12.9
51–60	30	15.5	17	15.9	12	14.1
>61	2	1.0	1	0.9	1	1.2

* Two respondents identified themselves as non-binary.

**Table 3:** Macular dystrophy/cone–rod dystrophy query results.

	All		Female		Male	
	*n*	%	*n*	%	*n*	%
Contacted without return of letters	281					
Response	194	69.0	107	55.2	85	43.8
***Vocational training response***	173	89.2	91	85.1	80	94.1
No problems	141	81.5	74	81.3	66	82.5
Not started	9	5.2	4	4.4	5	6.3
Unable to start desired training	17	9.8	9	9.9	7	8.8
Termination of desired training	3	1.7	2	2.2	1	1.3
Delay > 1 year	8	4.6	3	3.3	5	6.3
Changing more than twice	1	0.3	1	1.1	0	0.0
***Professional career responses***	157	80.9	86	80.4	69	81.2
No problems	109	69.4	58	67.4	50	72.5
Early retirement all	28	17.8	15	17.4	12	17.4
Early retirement > 40 years of age	23 (130)	17.7	12 (79)	15.2	10 (49)	20.4
Early retirement > 50 years of age	21 (102)	20.6	12 (62)	19.4	9 (39)	23.1
Changing more than twice	9	5.7	5	5.8	4	5.8
Unemployed > 1 year	15	9.6	10	11.6	5	7.2
***Social disadvantage responses***	190	97.9	106	99.1	82	96.5
None	53	27.9	30	28.3	22	26.8
Limitations in public life	104	54.7	59	55.7	44	53.7
Financial restrictions	35	18.4	19	17.9	16	19.5
Financial support acquired	54	28.4	29	27.3	24	29.3
Financial support sufficient	24 (54)	44.4	13 (29)	44.8	11 (24)	45.8
Financial support insufficient	30 (54)	55.6	16 (29)	55.2	13 (24)	54.2
***Family life disadvantage responses***	162	83.5	91	85.1	69	81.2
None	142	87.7	76	83.5	64	92.8
IRD reason for end of partnership	7	4.3	5	5.5	2	2.9
No children due to IRD	16	9.9	11	12.1	5	7.2
***Personal impairment responses***	190	97.9	107	100.0	81	95.3
None	23	12.1	12	11.2	11	13.6
Anxiety	141	74.2	81	75.7	58	71.6
Depression	30	15.8	15	14.0	14	17.3
Other	80	42.1	45	42.1	35	43.2
***Ophthalmic care responses***	190	97.9	105	98.1	82	96.5
Appropriate time	114	60.0	65	61.9	47	56.6
Delay all	65	34.2	34	32.4	31	37.8
Delay > 2 years	29	15.3	13	12.4	16	19.3
Delay > 5 years	36	18.9	21	20.0	15	18.1
Misdiagnosis	42	22.1	21	20.0	21	25.3
Insufficient psychological support	68	35.8	41	39.0	27	32.5

Italic and bold text indicates the summary of the repsonses for the subsections of the questionaire.

**Table 4:** Study characteristics and major differences between MD/CRD and RP patients.

	MD/CRD	RP *
*Study characteristics*		
Response rate	69.0%	67.2%
Number of responding patients	194	162
*Major differences*		
Under 51 years of age	47.4%	67.3%
Disease onset ≥ 40 years of age	37.6%	17.3%
Response rate in professional life	80.9%	88.3%
Early retirement all	17.8%	26.6%
Early retirement > 50 years of age	20.6%	50.0%
Sufficient social support	44.4%	60.0%
No negative impact on family life	87.7%	78.6%

* [19]. Italics are used as subheadings

## Data Availability

The data presented in this study are available upon request from the corresponding author due to legal restrictions.

## Supplementary Materials

The following supporting information can be downloaded at: https://www.mdpi.com/article/10.3390/jcm14227928/s1, Text S1: Questionnaire: original German version, Text S2: Questionnaire: translated English version.

## Abbreviations

The following abbreviations are used in this manuscript:

BDI	Beck Depression Inventory
CRD	Cone–rod dystrophy
HADS	Hospital Anxiety and Depression Scale
IRD	Inherited retinal dystrophy
MD	Macular dystrophy
RP	Retinitis pigmentosa
