# Supplementary material for: Patient-Reported Social Impact of Molecularly Confirmed Macular Dystrophies and Cone–Rod Dystrophies
Source: J Clin Med. 2025 Nov 8;14(22):7928. doi: 10.3390/jcm14227928 (PMC12653736; doi:10.3390/jcm14227928)
Supplement: Supplementary file 1 [file jcm-14-07928-s001.zip › jcm-3908004-supplementary.pdf]

Supplementary File S1
Fragebogen für Patienten mit Makuladystrophie/Zapfen-Stäbchendystrophie (MD/ZSD)

Sehr geehrte Patientin, sehr geehrter Patient,
wir bitten Sie diesen Fragebogen auszufüllen und bis zum 01.07.2024 im beigefügten Umschlag zurückzusenden. Sollte ein Teil der Fragen nicht auf Sie zutreffen oder sollten Sie einzelne Fragen nicht beantworten wollen, lassen Sie diese bitte offen, auch wenn Sie nur einen Teil der Fragen beantworten, unterstützen Sie diese Studie. Bei den Fragen 4-9 sind mehrere Antworten möglich.
Wir danken Ihnen für Ihre Unterstützung.
Mit freundlichen Grüßen
N. Zehe-Lindau Dr. G. Spital Prof. Dr. U. Kellner

1. Alter:
    - 11-20
    - 21-30
    - 31-40
    - 41-50
    - 51-60
    - 61-70

2. Geschlecht:
    - W
    - M
    - D

3. Alter beim Auftreten der ersten Symptome:
    - 0-10
    - 11-20
    - 21-30
    - 31-40
    - 41-50
    - 51-60
    - 61-70

4. Beeinträchtigung durch die Makuladystrophie (MD)/Zapfen-Stäbchendystrophie (ZSD) während meiner beruflichen Ausbildung:
    - Ich konnte meine gewünschte berufliche Ausbildung abschließen
    - Ich musste meine gewünschte und begonnene berufliche Ausbildung aufgrund der MD/ZSD abbrechen
    - Ich konnte meine gewünschte berufliche Ausbildung aufgrund der MD/ZSD nicht antreten
    - Ich habe meine berufliche Ausbildung wegen der MD/ZSD mehr als zweimal wechseln müssen
    - Ich habe wegen der MD/ZSD für meine berufliche Ausbildung mehr als ein Jahr länger benötigt als erwartet
    - Ich habe meine berufliche Ausbildung noch nicht begonnen

5. Beeinträchtigung durch die Makuladystrophie (MD)/Zapfen-Stäbchendystrophie (ZSD) während meiner beruflichen Tätigkeit:
   - Ich konnte meine berufliche Tätigkeit so lange wie geplant ausführen
   - Ich musste meine berufliche Tätigkeit aufgrund der MD/ZSD vorzeitig beenden
   - Ich konnte meine berufliche Tätigkeit nach der Ausbildung aufgrund der MD/ZSD nicht antreten
   - Ich habe meine berufliche Tätigkeit wegen der MD/ZSD mehr als zweimal wechseln müssen
   - Ich bin wegen der MD/ZSD mehr als ein Jahr arbeitslos gewesen
   - Ich bin wegen der MD/ZSD frühzeitig berentet worden

6. Soziale Beeinträchtigungen durch die Makuladystrophie (MD)/Zapfen-Stäbchendystrophie (ZSD):
   - Ich fühle mich nicht sozial beeinträchtigt aufgrund der MD/ZSD
   - Ich habe finanzielle Einschränkungen aufgrund der MD/ZSD
   - Ich fühle mich aufgrund der Einschränkungen durch die MD/ZSD in der Teilnahme am öffentlichen Leben eingeschränkt
   - Ich habe soziale (finanzielle oder andere unterstützende) Maßnahmen bisher nicht wahrgenommen
   - Ich habe soziale (finanzielle oder andere unterstützende) Maßnahmen wegen meiner RP wahrgenommen und empfinde diese als angemessen
   - Ich habe soziale (finanzielle oder andere unterstützende) Maßnahmen wegen meiner RP wahrgenommen und empfinde diese als unzureichend

7. Familiäre Beeinträchtigungen durch die Makuladystrophie (MD)/Zapfen-Stäbchendystrophie (ZSD)
   - Ich fühle mich in meiner familiären Situation nicht beeinträchtigt aufgrund der MD/ZSD
   - Ich habe eine Trennung von einem Partner erlebt aufgrund der MD/ZSD
   - Ich habe aufgrund der MD/ZSD auf eigene Kinder verzichtet

8. Persönliche Beeinträchtigungen durch die Makuladystrophie (MD)/Zapfen-Stäbchendystrophie (ZSD)
   - Ich fühle mich in meiner persönlichen Situation nicht beeinträchtigt aufgrund der MD/ZSD
   - Ich habe Angst in Bezug auf meine zukünftige persönliche Situation aufgrund der MD/ZSD
   - Ich habe Depressionen aufgrund der MD/ZSD
   - Ich habe andere persönliche Beeinträchtigungen aufgrund der R MD/ZSD

9. Beurteilung der augenärztlichen Versorgung bei Makuladystrophie (MD)/Zapfen-Stäbchendystrophie (ZSD)
   - Ich habe den Eindruck, dass die augenärztliche und molekulargenetische Abklärung meiner MD/ZSD in einem angemessenen Zeitraum erfolgt ist
   - Ich habe erst mehr als zwei Jahre nach meiner ersten augenärztlichen Untersuchung nach Symptombeginn die Diagnose MD/ZSD genannt bekommen
   - Ich habe erst mehr als fünf Jahre nach meiner ersten augenärztlichen Untersuchung nach Symptombeginn die Diagnose MD/ZSD genannt bekommen
   - Ich habe den Eindruck, dass ein oder mehrere Fehldiagnosen gestellt wurden, die die Erkennung meiner MD/ZSD verzögert haben
   - Ich vermisse eine psychologische Betreuung im Verlauf meiner MD/ZSD

Supplementary File S2
Survey for macular dystrophy or cone-rod dystrophy patients, translated version

Dear patients,
we kindly ask you to fill in this survey and return it until 01.07.2024 in the attached envelope. If some of the questions do not apply to or you choose not to respond to some of the questions, please let those be unanswered and respond to the other questions. Your answers are helpful, even if you only respond to some questions. For questions 4 -9 more than one answer is possible.
We are grateful for your support.
Best regards
N. Zehe-Lindau G. Spital, MD U. Kellner, MD

1. Age range:
   - 11-20
   - 21-30
   - 31-40
   - 41-50
   - 51-60
   - 61-70

2. Gender:
   - F
   - M
   - D

3. Age range at onset of first symptoms:
   - 0-10
   - 11-20
   - 21-30
   - 31-40
   - 41-50
   - 51-60
   - 61-70

4. Macular dystrophy (MD) or cone-rod dystrophy (CRD) related impairment during my vocational training:
   - I could complete my desired vocational training
   - I started, but had to terminate my desired vocational training due to MD/CRD
   - I could not start my desired vocational training due to MD/CRD
   - I had to change my vocational training more than twice due to MD/CRD
   - I needed one year longer than expected for my vocational training due to MD/CRD
   - I have not yet started my vocational training

5. Macular dystrophy (MD) or cone-rod dystrophy (CRD) related impairment during my professional career:
    - Ich konnte meine berufliche Tätigkeit so lange wie geplant ausführen
    - I had to terminate my professional career prematurely due to MD/CRD
    - I could not start my professional career after vocational training due to MD/CRD
    - I had to change my professional career more than twice due to MD/CRD
    - I have been unemployed for more than one year due to MD/CRD
    - I had to retire prematurely due to MD/CRD

6. Macular dystrophy (MD) or cone-rod dystrophy (CRD) related social impairment:
    - I do not feel socially impaired due to MD/CRD
    - I experience financial restrictions due to MD/CRD
    - I am restricted in my participation in public life due to MD/CRD
    - I have not obtained social support (financial or other means) so far
    - I have obtained social support (financial or other means) due to MD/CRD and consider the support as appropriate
    - I have obtained social support (financial or other means) due to MD/CRD and consider the support as inappropriate

7. Macular dystrophy (MD) or cone-rod dystrophy (CRD) related impairment of family life:
    - I do not feel impaired due to MD/CRD in my family life
    - I have experienced an end of partnership due to MD/CRD
    - I have abstained of children due to MD/CRD

8. Macular dystrophy (MD) or cone-rod dystrophy (CRD) related personal impairment
    - I do not feel impaired due to MD/CRD in my personal situation
    - I feel anxiety regarding my future personal situation due to MD/CRD
    - I experience depression due to MD/CRD
    - I experience other personal impairments due to MD/CRD

9. Assessment pf ophthalmic care for macular dystrophy (MD) or cone-rod dystrophy (CRD)
    - I feel, that the ophthalmologic and molecular genetic diagnostic process was performed within an adequate time period
    - I have received the diagnosis of MD/CRD more than two years after my first ophthalmologic examination at onset of my clinical symptoms
    - I have received the diagnosis of MD/CRD more than five years after my first ophthalmologic examination at onset of my clinical symptoms
    - I have the impression, that one or more misdiagnoses delayed the diagnosis of MD/CRD
    - I miss psychological support during the course of my MD/CRD
